# EEG decoding of spoken words in bilingual listeners: from words to language invariant semantic-conceptual representations

**DOI:** 10.3389/fpsyg.2015.00071

**Published:** 2015-02-06

**Authors:** João M. Correia, Bernadette Jansma, Lars Hausfeld, Sanne Kikkert, Milene Bonte

**Affiliations:** Department of Cognitive Neuroscience, Faculty of Psychology and Neuroscience, Maastricht Brain Imaging Center (M-BIC), Maastricht UniversityMaastricht, Netherlands

**Keywords:** EEG decoding, EEG oscillations, speech perception, spoken word recognition, bilinguals, semantic representations, conceptual representation

## Abstract

Spoken word recognition and production require fast transformations between acoustic, phonological, and conceptual neural representations. Bilinguals perform these transformations in native and non-native languages, deriving unified semantic concepts from equivalent, but acoustically different words. Here we exploit this capacity of bilinguals to investigate input invariant semantic representations in the brain. We acquired EEG data while Dutch subjects, highly proficient in English listened to four monosyllabic and acoustically distinct animal words in both languages (e.g., “paard”–“horse”). Multivariate pattern analysis (MVPA) was applied to identify EEG response patterns that discriminate between individual words within one language (within-language discrimination) and generalize meaning across two languages (across-language generalization). Furthermore, employing two EEG feature selection approaches, we assessed the contribution of temporal and oscillatory EEG features to our classification results. MVPA revealed that within-language discrimination was possible in a broad time-window (~50–620 ms) after word onset probably reflecting acoustic-phonetic and semantic-conceptual differences between the words. Most interestingly, significant across-language generalization was possible around 550–600 ms, suggesting the activation of common semantic-conceptual representations from the Dutch and English nouns. Both types of classification, showed a strong contribution of oscillations below 12 Hz, indicating the importance of low frequency oscillations in the neural representation of individual words and concepts. This study demonstrates the feasibility of MVPA to decode individual spoken words from EEG responses and to assess the spectro-temporal dynamics of their language invariant semantic-conceptual representations. We discuss how this method and results could be relevant to track the neural mechanisms underlying conceptual encoding in comprehension and production.

## Introduction

Speech processing is a surprisingly flexible and accurate cognitive ability that allows humans to comprehend spoken language in real-time. At the individual word level, speech processing requires a continuous mapping of complex and variable auditory input signals to words and their semantic-conceptual representations. In turn, when we speak, we start from ideas and concepts and convert these into articulatory motor programs. In multilingual environments, these transformations involve the extraction of unified semantic concepts from variable acoustic/phonological word forms in native and non-native languages. When and how the bilingual brain performs these language-invariant conceptual transformations remains essentially unknown and is a focus of the present electroencephalography (EEG) study.

EEG allows studying non-invasively and with high temporal resolution the neural dynamics of speech processing. The temporal dynamics of EEG signals are informative of temporal order effects during speech processing. ERP (event-related potential) components at early time intervals, 100–200 ms after word onset, have been associated with phonetic/phonological processing (Dumay et al., 2001; Sanders and Neville, 2003; Bonte and Blomert, 2004; Uusvuori et al., 2008). Intermediate time intervals (200–300 ms) have been suggested to reflect early aspects of lexical access (Van den Brink et al., 2001; Hagoort et al., 2004; Salmelin, 2007; Bonte et al., 2009), followed by lexical/semantic processing in the 300–600 ms window, as indicated by ERP modulations dependent on semantic attributes of words, semantic priming and semantic context (Kutas and Hillyard, 1980; Hagoort, 2008). Spatially, this temporal signature of speech processing may reflect a spread of information from primary auditory areas to anterior temporal and frontal regions, mid-inferior and posterior temporal regions (Marinkovic et al., 2003) corresponding to the network of brain areas observed in functional magnetic resonance imaging (fMRI) studies of speech processing (Binder et al., 2000; Hickok and Poeppel, 2007; Rauschecker and Scott, 2009). Complementary to ERP modulations, the oscillatory dynamics of EEG signals measured extracranially (Hagoort et al., 2004; Shahin et al., 2009; Doelling et al., 2014; Strauß et al., 2014) and intracranially (Luo and Poeppel, 2007; Giraud and Poeppel, 2012) have provided important insights regarding the function of underlying neural oscillations. Namely, an entrainment of theta band oscillations to the phoneme/syllable rate of speech signals, and the entrainment of gamma band oscillations to the phase of such theta band oscillations are suggested to reflect synchronization mechanisms that optimize the parsing of the speech signal into its relevant units (Lakatos et al., 2005; Giraud and Poeppel, 2012; Obleser et al., 2012; Peelle and Davis, 2012).

A challenge is to investigate how these temporal and oscillatory EEG dynamics encode the representation of specific speech units, such as individual words and concepts. Recently, methods based on machine learning comprising multivariate statistics (MVPA, multivariate pattern analysis, Formisano et al., 2008a; Haxby et al., 2011) have shown their potential to solve this challenge. MVPA of EEG signals extends traditional univariate methods by exploiting the interaction between multiple signal features (e.g., spectro-temporal features across multiple electrodes and/or time points) using classification algorithms (Chan et al., 2011b; Hausfeld et al., 2012; Herrmann et al., 2012; Brandmeyer et al., 2013). The higher sensitivity of MVPA to find information content within brain imaging signals has significantly contributed to our understanding of the brain's responses to speech and language. In fMRI studies, multi-voxel patterns across early and higher-order auditory cortex have been shown to successfully predict the (perceptual) identity of individual speech sounds and speaker's voices (Formisano et al., 2008b; Kilian-Hütten et al., 2011; Bonte et al., 2014). Furthermore, fMRI responses in inferior parietal areas have been shown to differentiate words across different semantic categories [e.g., tools and dwellings, Shinkareva et al. (2011)]. At a more fine-grained within-category level, MVPA was recently shown to accurately predict which spoken noun a bilingual listener was listening to in one language (e.g., “horse” in English) based on the fMRI response patterns to equivalent nouns in the other language (e.g., “paard” in Dutch; Correia et al., 2014). This generalization of the meaning of words across languages specifically relied on focal regions, including the left anterior temporal lobe (left-ATL), suggesting the existence of “hub” regions organizing semantic-conceptual knowledge in abstract form (Damasio et al., 1996; Scott et al., 2000; Patterson et al., 2007; Visser and Lambon Ralph, 2011; Correia et al., 2014). Although more challenging in terms of the robustness of single trial estimates, also spatially/temporally distributed EEG/MEG patterns have been observed to discriminate individual speech sounds (Hausfeld et al., 2012), and words from different perceptual and semantic categories (Simanova et al., 2010; Chan et al., 2011b; Sudre et al., 2012). Classification performances in EEG-MVPA studies on speech processing are typically low [e.g., below 0.55 in binary classification of spoken vowels, Hausfeld et al. (2012); or below 0.60 in binary classification of spoken words, Simanova et al. (2010)]. Besides the low signal-to-noise ratio of single trial EEG signals, EEG-based classification of individual words may be limited by the continuous and temporally variable processing of their phonological and semantic features (Van Petten et al., 1999). Importantly, however, multivariate approaches in EEG allow unraveling subtle differences in the neural processing of individual speech sounds that remain obscured in univariate approaches relying on average activation differences between experimental conditions.

**Figure 1:**
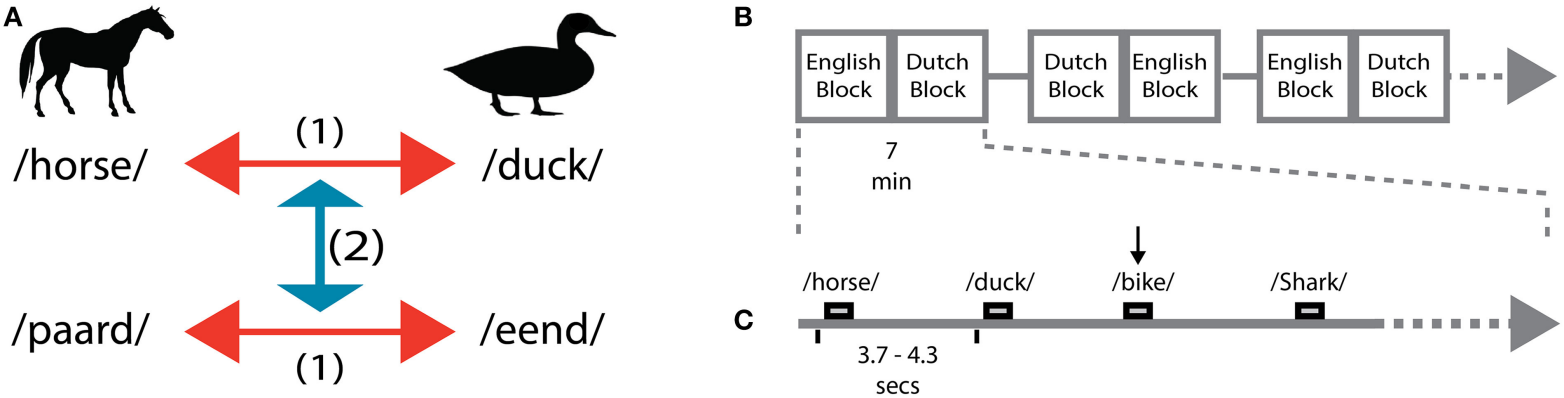
**Experimental design. (A)** Within-language discrimination (1) was performed for all pairwise comparisons in English and Dutch. Across-language generalization (2) was performed across translational equivalent words in the other language. Both generalization directions were performed, from English to Dutch and from Dutch to English. **(B)** Runs, blocks organization along the EEG session. Only 3 runs out of 8 runs are depicted for illustration. Each run (7 min) was composed by two blocks (English and Dutch). **(C)** Within each block, a jittered interval (ITI) of 3.7–4.3 s separates the presentation of the words. The black vertical arrow represents a response from the subjects to detect a non-animal word (e.g., bike).

Here, we employ MVPA to investigate spectro-temporal EEG response patterns capable of discriminating semantic-conceptual representations of words at the fine-grained level of within-category distinctions (animal nouns). To this end, we exploit the unique capacity of bilingual subjects to access semantic-conceptual information of spoken words from two languages. In separate Dutch and English blocks, we asked bilingual participants to listen to individual animal nouns (66.6% trials) and to detect non-animal target nouns (33.3% trials). The non-animal target nouns were presented as control task to ensure speech comprehension at every word presentation, but were not included in the analysis. Following supervised machine learning approaches, we trained multivariate classifiers (linear-SVM) to predict the identity of the perceived animal noun from new (untrained) samples of EEG activity (Figure 1A). In a first analysis we aimed to identify the EEG correlates involved in within-language word discrimination. To this end we trained classifiers to discriminate EEG responses to English (e.g., “horse” vs. “duck”) and Dutch (e.g., “paard” vs. “eend”) nouns. Importantly, stimuli included three exemplars of each noun, pronounced by three different female speakers, allowing for speaker-invariant word discrimination (“*within-language*”). In a second analysis we aimed to assess the EEG correlates involved in language-independent decoding of the animal nouns (“*across-language*”). Here we trained classifiers to discriminate EEG responses to words in one language (e.g., in English, “horse” vs. “duck”) and tested whether this training generalizes and allows discrimination of EEG responses to the corresponding nouns in the other language (e.g., in Dutch, “paard” vs. “eend”). Importantly, all words were acoustically-phonetically distinct both within and across languages. Based on this approach, we aimed to investigate whether language-independent representations are detectable in the EEG responses to individual spoken words. In particular, this approach allowed us to extract critical time windows and frequency ranges within the EEG relevant to semantic-conceptual encoding.

## Methods

### Participants

Sixteen native Dutch (L1) participants proficient in English (L2) took part in the study (8 males and 8 females, right-handed, mean age = 28.9 SD = 3.4). The participants were undergraduate or post-graduate students of Maastricht University studying or working in an English speaking environment. All participants reported normal hearing abilities and were neurologically healthy. English proficiency was assessed with the LexTALE test, a vocabulary test including 40 frequent English words and 20 non-words (Lemhöfer and Broersma, 2012). The mean test score was 89.6% correct (SD = 11.2%). This score is well above the average score (70.7%) of a large group of Dutch and Korean advanced learners of English performing the same test (Lemhöfer and Broersma, 2012). For comparison reasons, participants also conducted the Dutch version of the vocabulary test. The mean Dutch proficiency score was 94.1% (SD = 3.3). The study was approved by the Ethical Committee of the Faculty of Psychology and Neuroscience at the University of Maastricht, The Netherlands.

### Stimuli

Stimuli consisted of Dutch and English spoken words representing four different animals (English: “Bull,” “Duck,” “Horse,” and “Shark,” and the Dutch equivalents: “Stier,” “Eend,” “Paard,” and “Haai”) and six inanimate object words (English: “Bike,” “Coat,” “Dress,” “Road,” “Suit,” and “Town”; and the Dutch equivalents: “Fiets,” “Jas,” “Jurk,” “Weg,” “Pak,” and “Stad”). All animal nouns were monosyllabic and acoustically/phonetically distinct from each other both within and across languages. Phonetic distance between word pairs was quantified using the Levenshtein distance, which gives the number of phoneme insertions, deletions and/or substitutions required to change one word into the other, divided by the number of phonemes of the longest word (Levenshtein, 1965). On a scale from 0 (no changes) to 1 (maximum number of changes), the mean (SD) Levenshtein distances corresponded to 0.83 (0.15) for Dutch word pairs, 0.93 (0.12) for English word pairs and 1.00 (0.00) for English-Dutch word pairs. Furthermore, all animal nouns had an early age of acquisition in Dutch (mean = 5.28 years SD = 0.98; De Moor et al., 2000) and a medium-high frequency of use expressed on a logarithmic scale in counts per million tokens in Dutch (mean = 1.29 SD = 0.71) and in English [mean = 1.50 SD = 0.42; Celex database, Baayen et al. (1995)]. To add acoustic variability and allow for speaker-invariant MVPA analysis, the words were spoken by three female native Dutch speakers with good English pronunciation. Stimuli were recorded in a sound proof chamber at a sampling rate of 44.1 kHz (16 bit resolution). Post-processing of the recorded stimuli was performed in PRAAT software (Boersma and Weenink, 2013) and included band-pass filtering (80–10,500 Hz), manual removal of acoustic transients (clicks), length equalization, removal of sharp onsets and offsets using 30 ms ramp envelopes, and amplitude equalization (average RMS). Stimulus length was equated to 600 ms (original range: 560–640 ms) using PSOLA (75–400 Hz as extrema of the F0 contour). We carefully checked the stimuli for possible alterations in F0 after length equation and did not find any detectable changes. We assured that the produced stimuli were unambiguously comprehended by the participants during the stimuli familiarization phase prior to the experiment.

### Experimental procedures

The experimental session was organized in 8 runs, each run containing 2 blocks (one Dutch and one English). Each block included 36 nouns: 24 animal nouns and 12 (33.3%) non-animal nouns. The order of English and Dutch blocks was counterbalanced across runs: odd runs started with an English block followed by a Dutch block; even runs started with a Dutch block followed by an English block (Figure 1B). Participants were instructed to actively listen to the stimuli and to press a button (with the left index finger) whenever they heard a non-animal word. The goal of the task was to help maintaining a constant attention level throughout the experiment and to promote speech comprehension at every word presentation. All participants paid attention to the words as indicated by a mean (SD) detection accuracy of 98.3 (1.4) %. Data from non-animal trials were excluded from further analysis. The 24 animal nouns in each block corresponded to 6 repetitions of each of the 4 animal nouns. Because nouns were pronounced by 3 different speakers, each physical stimulus was repeated twice in each block. Stimulus presentation was pseudo-randomized within each block, avoiding consecutive presentations of the same words or sequences of words. Throughout the experiment, each animal noun was presented 48 times per language.

### EEG acquisition and preprocessing

Data were recorded with a sampling rate of 250 Hz in an electrically shielded and sound-proof room from 62 electrode positions (Easycap, Montage Number 10, 10–20 system) relative to a left mastoid reference signal. The ground electrode was placed on the Fz electrode. Impedance levels were kept below 5 kΩ. During the EEG measurement, stimuli were presented binaurally at a comfortable intensity level. According to an event-related design (Figure 1C), the averaged inter-trial-interval between two stimuli was 4 s (jittered randomly between 3.7 s and 4.3 s). Each run took 7 min, resulting in a total EEG measurement time of 56 min. A gray fixation cross against a black background was used to keep the visual stimulation constant during the whole duration of a block. Block and run transitions were marked with written instructions. Participants were instructed to minimize eye-movements during the auditory presentation and fixate on the fixation cross.

Data preprocessing was performed using EEGlab (Delorme and Makeig, 2004) and included band-pass filtering (0.1–100 Hz) followed by epoch extraction locked to the onset of the animal nouns (−1000 to 1000 ms) and baseline correction (−1000 to 0 ms).

Removal of signal artifacts was performed in two steps. First, the data were visually inspected and epochs containing non-stereotypical artifacts including high-amplitude, high-frequency muscle noise, swallowing, and electrode cable movements, were rejected (mean 4.31 trials per subject, SD 2.36). Second, stereotypical artifacts related to eye movements, eye-blinks and heart beat artifacts were corrected with extended INFOMAX ICA (Lee et al., 1999) as implemented in EEGLAB. Because data were recorded at 62 channels, runica decomposed the data in 62 component activations per subject. These component activations were categorized as EEG activity or non-brain artifacts by visual inspection of their scalp topographies, time courses, and frequency spectra. Criteria for categorizing component activations as EEG activity included (1) a scalp topography consistent with an underlying dipolar source, (2) spectral peak(s) at typical EEG frequencies, and (3) regular responses across single trials, i.e., an EEG response should not occur in only a few trials (Delorme and Makeig, 2004). Based on these criteria, component activations representing non-brain artifacts were removed, and EEG data were reconstructed from the remaining component activations representing brain activity. The resulting ICA-pruned data sets were baseline corrected (–1000 to 0 ms) and used for further analysis.

### ERP and ERSP analysis

First, in order to validate typical EEG responses to spoken words reported in the literature, we performed univariate analyses. These were conducted in EEGlab (Delorme and Makeig, 2004) and included: (1) an ERP analysis based on the average amplitude of signal change over time with respect to baseline (−1000 to 0 ms) and (2) an ERSP (event-related spectral perturbation) analysis based on averaged power changes of all words over frequency and time with respect to baseline (−1000 to 0 ms). For the ERSP analysis we employed a Hanning taper fast fourier transform (FFT) filter from 1 to 60 Hz on a linear frequency scale with steps of 2 Hz, producing 30 filtered signals. Group statistics for the ERP and ERSP were performed at random-effects using two-sided Wilcoxon tests for each time-point vs. zero baseline and corrected for multiple comparisons using FDR (alpha = 5%).

### Multivaritate classification analysis

Multivariate classification was employed to investigate whether specific temporal or spectrotemporal EEG signatures enable the discrimination of words within and across languages. To this end we used a supervised machine learning algorithm (linear support vector machines, linear-SVM; Cortes and Vapnik, 1995) as implemented by the Bioinformatics Matlab toolbox (maximum number of learning iterations = 15,000). Classifications were performed to evaluate whether patterns of EEG data pertained relevant information encoding the representations of spoken words (within-language discrimination) as well as their language invariant semantic-conceptual representations (across-language generalization). All classifications were binary (i.e., chance-level is 0.5) and involved discrimination and generalization between two words. The results of these binary predictions were then averaged across all possible pair-wise classifications. Additional methodological steps encompassing the computational strategy to validate the classification results (cross-validation) and to select the EEG features used for classification (feature selection) are described below.

### Cross-validation approaches

Cross-validation of the multivariate classification analysis served two purposes: (1) to obtain robust estimates of the discrimination accuracies; (2) to allow generalization of classes by using distinct class groupings during the training and testing phases of classification. Cross-validation for within-language word discrimination relied on speaker identity. Here, we trained a classifier to discriminate words based on samples recorded from two out of the three speakers that pronounced the words (32 trials per word) and tested whether this training was able to generalize the left-out speaker pronouncing the same words (16 trials per word). This cross-validation procedure assured word discrimination invariant to neural activations specific to acoustic-phonetic characteristics of the speakers. Cross-validation for across-language generalization of semantic concepts relied on language independent information of the words. Here, we trained a classifier to discriminate words within one language (48 trials per word) and tested whether this training generalized to the other language (48 trials per word). Hence, in across-language generalization, we aimed to isolate semantic conceptual properties of the words that were language invariant.

### Feature selection approaches

#### Temporal-windows approach (shifting-windows + all channels)

**Figure 2:**
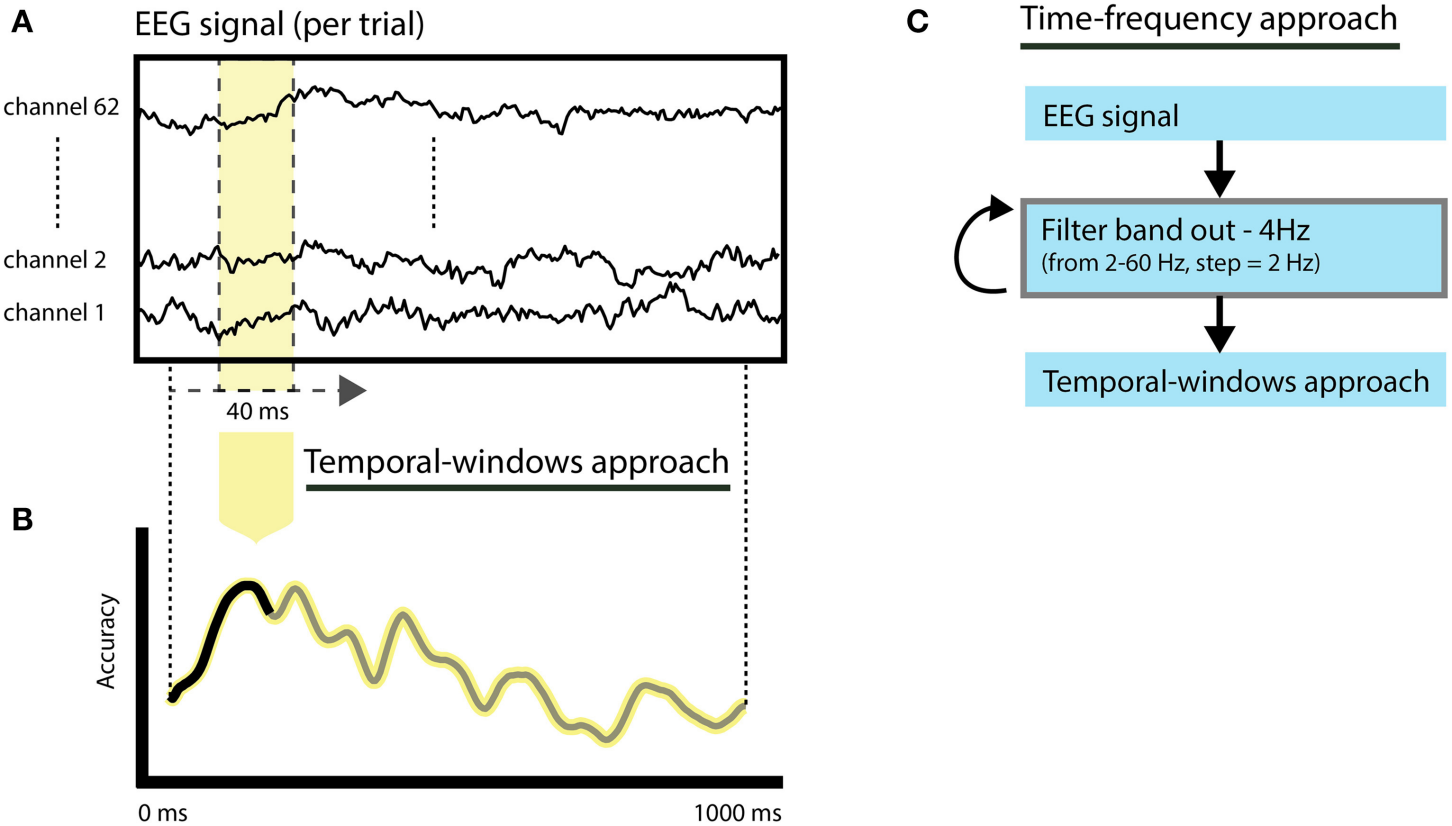
**Illustration of the feature selection approaches. (A)** The original epoched EEG response per word corresponds to the signal of all EEG channels and the interval from 0 to 1000 ms after word onset. **(B)** Temporal-windows approach. Classification relies on temporal windows of 40 ms (10 time-points) and all channels, resulting in classification accuracies over time. **(C)** Time-frequency approach. Thirty leave-band-out filtered versions of the signal are created (from 2 to 60 Hz, band-width = 4 Hz) following classification based on the same procedure employed in the temporal-windows approach.

To investigate the temporal evolution of spoken word decoding, we selected EEG response features (Figure 2A) using shifting-windows (width = 40 ms—10 time points) across all channels (Figure 2B). Restricting the EEG signal features to specific time windows permits the calculation of changes in classification accuracies over time informative of spoken word processing. Because the temporal-windows approach reduces the number of EEG features used for classification, it increases the temporal sensitivity of the classifiers to speaker and language invariant information of the spoken words due to a potentially better match between the training and testing patterns (Hausfeld et al., 2012). Additionally, it reduces the high dimensionality of the feature space, thus avoiding degraded classification performances (model overfitting; for a description, see Norman et al., 2006). The empirical null distribution was computed per subject using 200 label permutations. Individual statistical significance (*p* < 0.05) was calculated based on deviance from permuted accuracies. Group level statistics were calculated based on the overlap of significant subjects across time intervals using a binomial test with *n* = 16 (number of subjects) and *p* = 0.05 (Darlington and Hayes, 2000; Hausfeld et al., 2012) and corrected for multiple comparisons (time windows) using FDR correction (alpha = 5%).

#### Time-frequency approach (filtered-band-out + shifting-windows + all channels)

To assess the importance of brain oscillations in specific frequency bands to the performance of the classifiers we employed a feature selection approach combining temporal shifting windows and filter-band-out (Figure 2C). The original epoched EEG responses (−1000 to 1000 ms) were filtered prior to classification using an FIR (finite impulse response) filter as implemented in EEGlab (Delorme and Makeig, 2004). The width of the filtered-out frequency band was set to 4 Hz, centered on frequencies from 2 up to 60 Hz and in frequency steps of 2 Hz, producing 30 filtered signals. For each of the filtered signal versions, we subsequently performed the *temporal-windows* approach to assess the importance of each frequency band over time. The importance of the left-out frequency band was quantified in terms of a change in classification performance with respect to the non-filtered signal. To prevent a modulation of time-frequency importance due to differences in the original classification accuracy, a normalization of the importance of each time-frequency bin with respect to the accuracy limits (0–1) was performed using “odds-ratio” normalization (Szumilas, 2010). Odds-ratio values above 1 indicate a reduction of classification accuracy after a specific frequency band is filtered out. This approach allowed us to investigate the contribution of each frequency band over time without disrupting EEG spectral interactions that may be crucial in many cognitive processes, including speech processing (Giraud and Poeppel, 2012; Henry and Obleser, 2012; Peelle and Davis, 2012). Group statistics were performed in random-effects (two-sided Wilcoxon's test) and corrected for multiple comparisons using FDR correction (alpha = 5%).

## Results

### ERPs and time-frequency analysis

**Figure 3:**
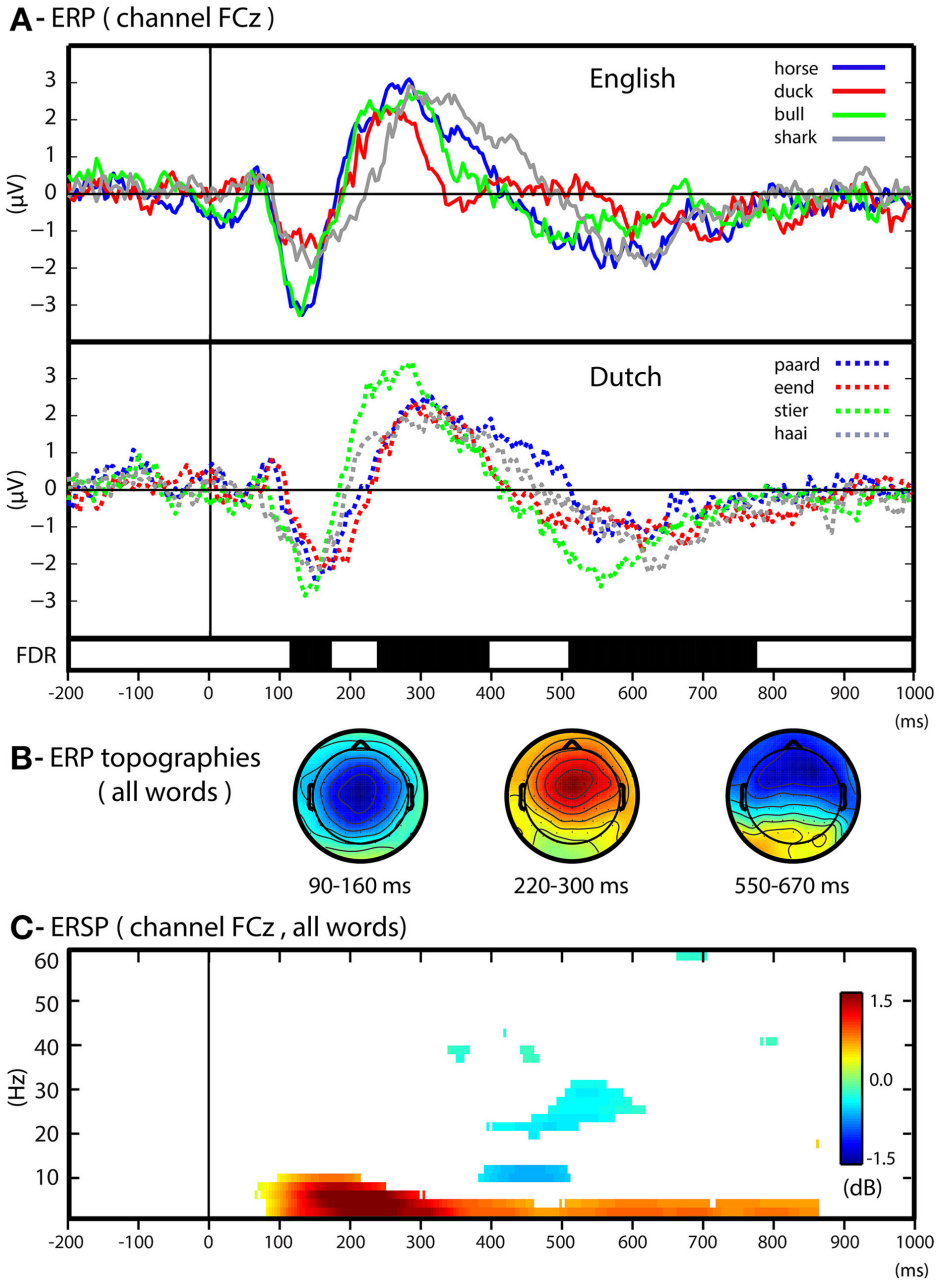
**Univariate results. (A)** ERP in respect to baseline of each word over the channel FCz. The ERPs for English and Dutch words are plotted separately. Group level statistics of all words with respect to baseline (Wilcoxon's test, FDR corrected < 0.05) is depicted in black bars during the time course of the ERP responses. **(B)** ERP scalp maps for time-intervals characteristic of the ERP components (N1: 90–160; P2: 220–300; N400: 550–670). **(C)** ERSP (dB) with respect to baseline for all words. The ERSP time-frequency plot includes a statistical threshold for group level significance (Wilcoxon's test in respect to baseline period, FDR correction, alpha = 0.05).

We first conducted univariate analyses of ERP and time-frequency changes relatively to stimulus baseline in order to assess the overall spectro-temporal characteristics of EEG responses evoked by the animal words. Figure 3 illustrates the averaged ERP responses elicited by the different animal words, including the expected ERP peaks (channel Fcz, Figure 3A) and their corresponding topographies (Figure 3B), in the N1 window (120–160 ms), the P2 window (230–390 ms) and the N400 window (550–800 ms). To assess univariate differences between the ERP responses we conducted all possible word-to-word contrasts within the same language (e.g., horse vs. duck), as well as all possible concept-to-concept contrasts (e.g., horse + paard vs. duck + eend). None of the possible contrasts yielded significant differences within or across participants.

The analysis of averaged power changes in different frequency bands (Figure 3C) shows an average power increase (ERS, event-related synchronization) of slow oscillations (1–10 Hz) starting 100 ms after stimulus onset, followed by a steep reduction in alpha power (ERD, event-related desynchronization) between 400 and 500 ms. At later time intervals, the ERS of slow oscillations (1–8 Hz) was maintained. These differences did not allow the systematic discrimination of individual words nor of language-independent concepts.

### Multivariate analysis (MVPA)

The multivariate analysis consisted of assessing the ability of multivariate classifiers to discriminate words within the same language and across first and second language in bilingual subjects. To assess the contribution of specific EEG features used for classification we used two feature selection approaches: a *temporal-windows approach*, relying on restricted time intervals (40 ms) shifted over time and all EEG channels; and a *time-frequency approach*, relying on a combined selection of features using the *temporal-windows approach* and a moving filter-band-out procedure (4 Hz bands with an step of 2 Hz).

**Figure 4:**
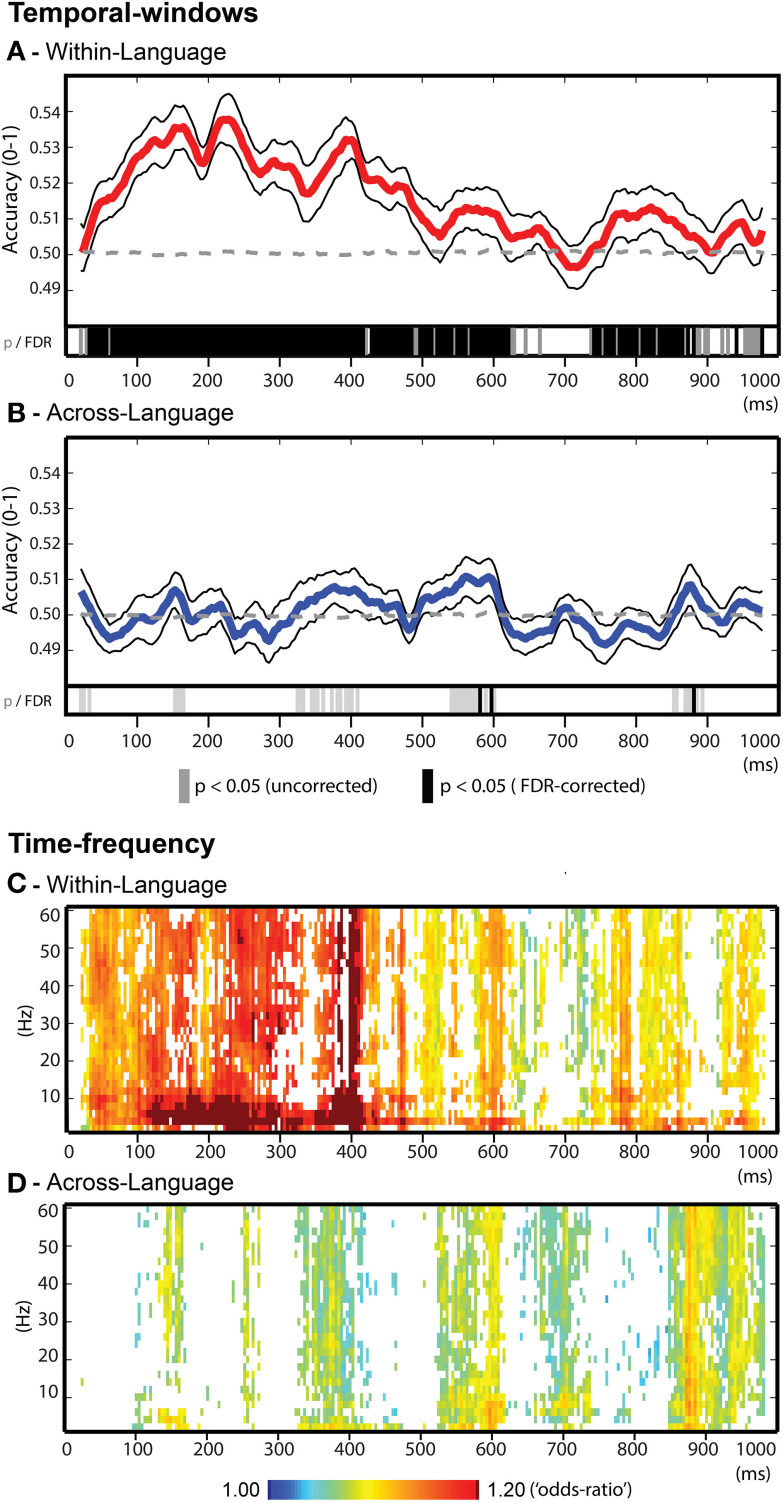
**Decoding results for the temporal-windows and time-frequency feature selection approaches. (A)** Temporal-windows approach for within-language discrimination. Group average accuracy time-course depicted in red line, the black lines represent one standard error above and below the average accuracy. **(B)** Temporal-windows approach for across-language generalizations. Group average accuracy time-course depicted in blue line, upper and lower standard errors in black lines. **(A–B)** Statistical results are reported at the group level (binomial test, *p* < 0.05) in gray bars and in black bars after FDR correction (alpha = 5%). **(C)** Time-frequency approach for within-language discrimination. **(D)** Time-frequency approach for across-language generalization. **(C–D)** Results are reported as averaged “odds-ratio” values at the group level (scaled between 1 and 1.2) and threshold using Wilcoxon's test following FDR correction (alpha = 5%).

The *temporal-windows* feature selection approach enabled identifying specific time-intervals related to word decoding. Within-language discrimination (Figure 4A) was significantly possible throughout most of the time-course from ~50 until 620 ms after word onset. Within this broad time window, salient local maxima of accuracies were identified for the temporal windows (40 ms) around 160 ms (accuracy = 0.535), 225 ms (accuracy = 0.537), 390 ms (accuracy = 0.533), 570 ms (accuracy = 0.513), and 820 ms (accuracy = 0.512). Interestingly, across-language generalization (Figure 4B) led to significant classification in more restricted temporal windows with significant results between 550 and 600 ms (maximum accuracy = 0.511) and 850–900 ms (maximum accuracy = 0.508). A further time-interval showing a trend (uncorrected *p* < 0.05) for across-language generalization capacity was observed around 400 ms (maximum accuracy = 0.507).

The *time-frequency* feature selection approach assessed the contribution of oscillatory activity in specific frequency bands to word decoding across the different time windows. For this purpose, “odds-ratio” values were computed, group averaged and thresholded for statistical significance (random-effects, FDR = 5%). Overall, the temporal profiles of the *time-frequency approach* match consistently with that of the *temporal-windows approach*, confirming that reductions in classification accuracy due to the omission of specific frequency bands occurred in time windows relevant for word decoding (Figure 4C). For within-language discrimination of words, reductions in classification accuracy especially occurred when omitting slow oscillations (below 12 Hz, delta, theta and alpha). For across-language generalization (Figure 4D), the period around 600 ms that showed significant generalization capacity, was characterized by accuracy reductions when filtering out frequencies up to 10 Hz (delta-theta-alpha). In other time windows a contribution of slow oscillations was also observed for this analysis, although involving slower oscillations (delta/ low theta, below 6 Hz). Visual inspection of Figures 4C–D further suggested that besides the sensitivities for oscillations below 12 Hz, for both types of analysis smaller classification drops occurred across gamma band (above 30 Hz) as well as across broad-band oscillation profiles.

## Discussion

By combining EEG MVPA and an experimental design that exploits the unique capacities of bilingual listeners we identified specific time windows and oscillations enabling within-category discrimination of individual spoken words. We demonstrated within-language word decoding in a broad time-window from ~50 to 620 ms after word onset with a strong contribution of slow oscillations (below 12 Hz). Most importantly, we were able to isolate specific time windows, including the 550–600 ms window, in which EEG features enabled the generalization of the meaning of the words across their Dutch and English word forms. Our results demonstrate the feasibility of using MVPA to identify individual word representations based on speech evoked EEG signals. Furthermore, they indicate the advantage of feature selection approaches in assessing temporal and temporal-oscillatory EEG response features in classification.

The univariate analyses illustrate ERP and oscillatory responses typically elicited by individual spoken words (Kutas and Federmeier, 2000; Hagoort et al., 2004; Bastiaansen et al., 2008; Bonte et al., 2009; Strauß et al., 2014) indicating a progression from acoustic-phonetic to lexical-semantic processing. The ERPs to the individual words show variability as a consequence of acoustic-phonetic differences and other word-specific properties. However, these differences did not allow the systematic discrimination of individual words nor of language-independent concepts. The prevalence of slow oscillatory activity (below 12 Hz) while subjects listened to the words indicates the crucial role of these frequencies in the processing and comprehension of speech (Hagoort et al., 2004; Giraud and Poeppel, 2012; Strauß et al., 2014). The analysis also showed that the univariate frequency power changes were not suitable for distinguishing individual words or across-language generalization of semantic concepts.

Importantly, the multivariate analyses allowed finding neural time-course correlates of the individual words that were invariant to the acoustic-phonetic characteristics of the speakers (within-language discrimination) as well as to the language in which the meaning was presented (across-language generalization). Within-language word discrimination relied on acoustic-phonetic and semantic-conceptual differences between the nouns, but also on possible other differences reflecting their individual properties. Accordingly, within-language discrimination was possible for both approaches of feature selections employed. In the *temporal-windows* approach (Figure 4A), investigating the temporal evolution of classification across consecutive short time-intervals of 40 ms, classification performance was significant from ~50 until 620 ms after word onset. In accordance with the ERP literature, decoding in this broad time window may be reflect a progression from phonetic-phonological processing (100–200 ms; Dumay et al., 2001; Sanders and Neville, 2003; Bonte and Blomert, 2004; Uusvuori et al., 2008) to initial lexical access (200–300 ms; Van den Brink et al., 2001; Hagoort et al., 2004; Salmelin, 2007; Bonte et al., 2009), and lexical semantic processing (300–600 ms; Kutas and Hillyard, 1980; Hagoort, 2008). These results are also consistent with previous single trial auditory word classification (Simanova et al., 2010) that showed initial prominent classification capability centered around 240 ms followed by a second less prominent capability around 480 ms after word onset.

The second multivariate analysis - across-language generalization - relied uniquely on language invariant semantic-conceptual properties of the nouns. This analysis, and especially the temporal-window approach (Figure 4B), revealed language invariant EEG features coding for the animal words in much more restricted time-windows including the 550–600 ms window and the 850–900 ms window at the end of the EEG epoch. ERP research has commonly associated similar time intervals with lexical-semantic processing of words across different task and sentence contexts (Kutas and Federmeier, 2000; Hagoort, 2008). Here, we indicate the potential of EEG signals to represent semantic-conceptual information of individual words independent of their acoustic-phonetic implementation or word-form. In order to isolate these input-invariant lexical-semantic representations we used animal nouns that were acoustically-phonetically distinct both within and across languages and were presented together with non-animal nouns that served as targets. In everyday speech processing, it is more difficult to disentangle input-driven vs. input-independent processes as initial lexical-semantic access is influenced by both acoustic-phonetic word form information (McClelland and Elman, 1986; Marslen-Wilson, 1987) and semantic or task context (Bonte, 2004; Obleser et al., 2004; Çukur et al., 2013), leading to early lexical and/or semantic ERP modulations around 200–300 ms (e.g., Van den Brink et al., 2001; Bonte et al., 2006; Travis et al., 2013; Strauß et al., 2014). Our approach presents a way to disentangle these aspects of comprehension. Importantly, by using words belonging to the same semantic category—animals—we reduced the influence of larger scale semantic category differences that can also drive the decoding of individual nouns (Simanova et al., 2010; Chan et al., 2011b; Shinkareva et al., 2011).

In later time-windows, significant classification for within-language discrimination (750–900 ms) and across-language generalization (850–900 ms) may reflect effects specific to our paradigm. That is, the slow presentation of words and/or the use of a target detection task, may have led to e.g., subvocal rehearsal in working memory (Kutas and Federmeier, 2000; Baddeley, 2003; Buchsbaum et al., 2011) and/or response monitoring toward the end of the trial (Wang et al., 2012).

In bilinguals, the active translation of written words during speech production tasks has been shown to elicit ERP differences for translation direction around 400 ms after word presentation (Christoffels et al., 2013). In the current study the effect of direct translations was minimized in several ways. First, we avoided active translations from second to native language and vice-versa by separately presenting words in Dutch and English blocks and using catch trials consisting of Dutch and English non-animal words, respectively. Furthermore, we used a selection of words with relatively early age of acquisition and of medium-high frequency of use in both languages.

To further understand the EEG temporal patterns allowing classification, we employed a time-frequency feature selection approach that assessed the relative contribution of oscillatory bands. We observed a significant contribution of slow EEG oscillations (below 12 Hz) for within-language and across-language classification, which links to the synchronization of oscillatory bands observed in the ERSP analysis. Furthermore, in the time windows during which the slower oscillations most strongly influenced classification performance, results also indicated a contribution from higher, gamma band oscillations (above 30 Hz). It would be interesting to replicate this possible co-occurrence of slower and gamma band modulations in future studies with bilinguals, and, in particular to test how they relate to suggested processing of (phonemes, syllables and semantic information (Lakatos et al., 2005; Giraud and Poeppel, 2012; Peelle and Davis, 2012; Peña and Melloni, 2012).

We may hypothesize that the neural processing underlying the EEG-based translations of animal nouns occurs in a brain network that was recently identified in an fMRI study using a comparable bilingual paradigm (Correia et al., 2014). In particular, in this previous study, language-invariant classification of animal words was found to rely on focal brain regions, including the left anterior temporal lobe (left-ATL), corroborating the existence of “hub” regions organizing semantic-conceptual knowledge in abstract form. Correspondingly, recent models of conceptual knowledge (Patterson et al., 2007), brain lesion studies (Damasio et al., 1996) and neuroimaging evidence (Visser et al., 2012; Correia et al., 2014) locate a possible semantic hub within the left-ATL, integrating distributed semantic-conceptual information throughout the cortex. Furthermore, distributed neural representations of semantic information may also connect to modality specific brain regions subserving perception and action (Martin, 2007; Meyer and Damasio, 2009). Interestingly, magnetoencephalography (MEG) studies have related time windows starting at 400 ms after spoken word onset to semantic processing in bilateral anterior temporal areas (Marinkovic et al., 2003; Chan et al., 2011a), suggesting a putative link between the present finding of language-independent word decoding in the 550–600 ms time window and processing in these brain regions. At present, this spatial-temporal association remains speculative, but similar classification paradigms using simultaneous fMRI and EEG recordings (De Martino et al., 2011) may allow investigating the joint spatio-temporal representation of spoken words. Furthermore, earlier indications of semantic/conceptual representations of our words are observed in a spread time window between 320 and 420 ms after word onset (uncorrected *p* < 0.05). These and possibly even earlier semantic activations elicited by the individual animal words may be more difficult to detect due to variability in the exact timing of these initial activations.

Overall, our results show the benefit of EEG-based MPVA to investigate the representation of semantic concepts independently of the input language and more generally of individual spoken words independently of the speaker. Although the obtained accuracies are relatively low, they demonstrate the sensitivity of multivariate classification to distinguish subtle representations extracted from single-trial EEG responses that may not be present in the averaged EEG signal across multiple trials (Makeig et al., 2002; Hausfeld et al., 2012). Furthermore, our results show the potential of feature selection approaches based on moving temporal windows to highlight time windows associated with the neural processing of specific characteristics of speech and language (e.g., language independent semantic processing, see also Simanova et al., 2010; Chan et al., 2011b; Hausfeld et al., 2012). Future studies including different sets of words, languages or feature selection approaches may help confirming the generalization of our results. Beyond decoding language-invariant semantic-concepts during listening, EEG-based MVPA may also be used to investigate whether semantic-concepts share a similar neural representation during reading and speaking (Hickok et al., 2011; Pickering and Garrod, 2013). When we speak, we start from ideas and concepts and convert these into articulatory motor programs. ERP studies on speech production (e.g., picture naming), relate early windows, 100–200 ms after stimulus onset to interactive processing of visual encoding and accessing concepts for language use (Rahman and Sommer, 2003; Redmann et al., 2014). Like in speech comprehension, this interaction between input-dependent and abstract semantic-conceptual representations in speech production, together with their strong context and task-dependency (e.g., Jescheniak et al., 2002; Aristei et al., 2011), makes it difficult to isolate abstract semantic conceptual representations using univariate analysis methods. Because our EEG-based MVPA approach may disentangle these processes, it would thus be of interest to employ this same approach in speech production studies (e.g., and Schmitt et al., 2000; Koester and Schiller, 2008). In particular, a similar bilingual paradigm involving word naming in bilingual speakers would allow investigating the timing of language-independent semantic-conceptual representations. Furthermore, the classification of spoken words across and within languages in bilingual speakers and across and within speech modality (perception and production) may allow to investigate neural representations crucial for the initiation of speech production (Levelt, 1989; Rahman and Sommer, 2003; Indefrey and Levelt, 2004; Indefrey, 2011), as well as, for the monitoring of speech output (Hickok et al., 2011).

### Conflict of interest statement

The authors declare that the research was conducted in the absence of any commercial or financial relationships that could be construed as a potential conflict of interest.
